# Supplementary material for: Attractor dynamics with activity-dependent plasticity capture human working memory across time scales
Source: Commun Psychol. Author manuscript; Available in PMC 2024 May 17. (PMC11101211; doi:10.1038/s44271-023-00027-8)
Supplement: Supplementary Material [file NIHMS1985102-supplement-Supplementary_Material.pdf]

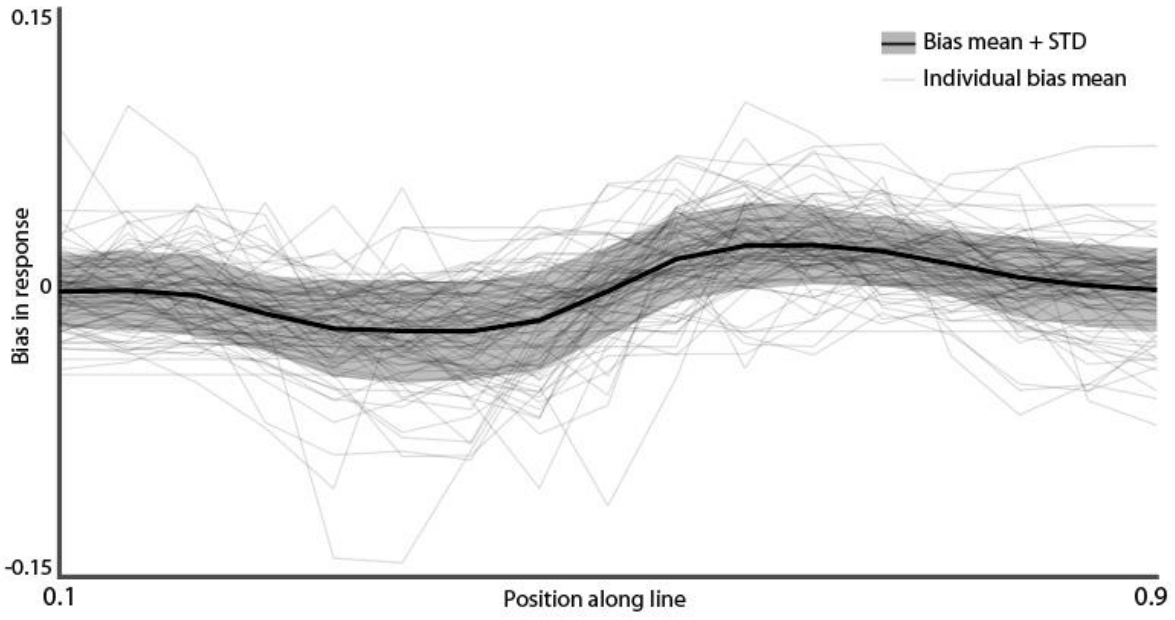

Supplement Figure 1: ***Individual differences in error distributions at 6s delay.*** *Thin lines show bias in response as a function of position along the line for each individual subject. Thick line and shaded area show mean and standard deviation of mean bias across subjects.*

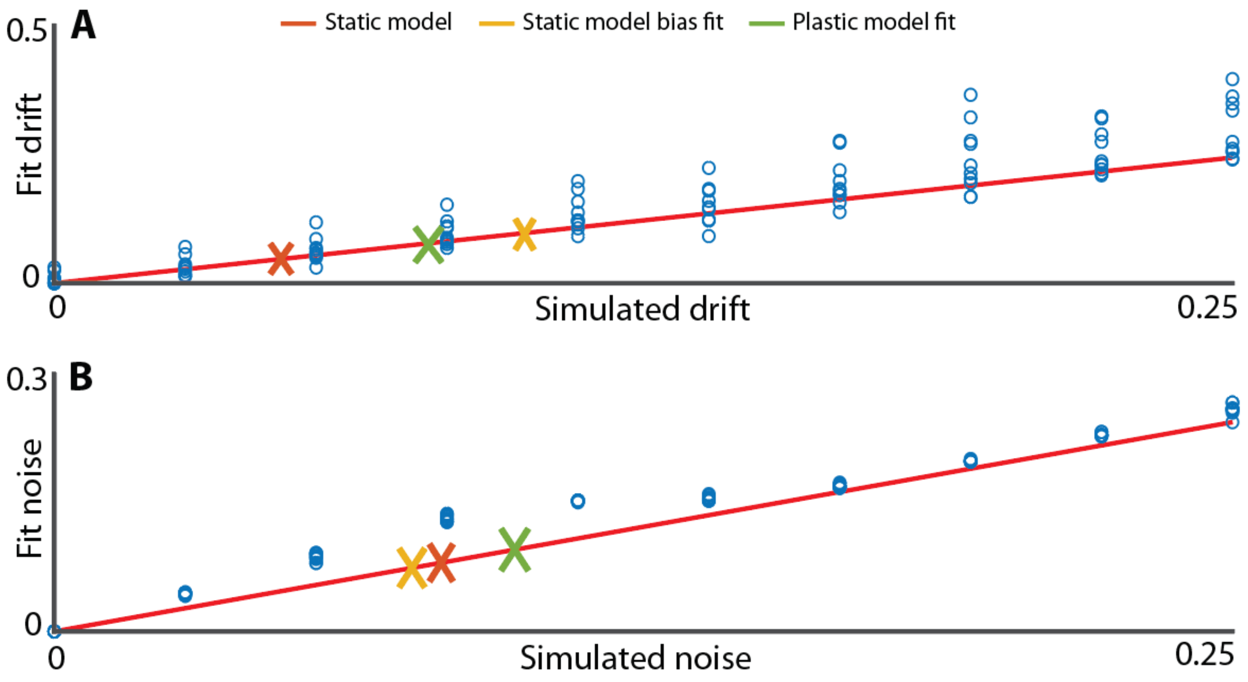

Supplement Figure 2: ***Fit variability of drift-diffusion model****. Diffusion on an energy landscape is simulated at various levels of drift strength and noise variance. The simulated data is then fit using our fitting procedure (Methods).* ***A)*** *Blue dots show simulated drift strength vs fit drift strength. The red line has a slope of 1 and an intercept of 0. X's indicate the estimates of the parameters of the model fits to experimental data. The orange X shows the fit value for the static model, the yellow X shows the fit value for the static model fit to the bias and the green X shows the fit value for the plastic model.* ***B)*** *Blue dots show simulated noise variance strength vs fit noise variance. The red line has a slope of 1 and an intercept of 0. The orange X shows the fit value for the static model, the yellow X shows the fit value for the static model fit to the bias and the green X shows the fit value for the plastic model, all reported in the main text.*

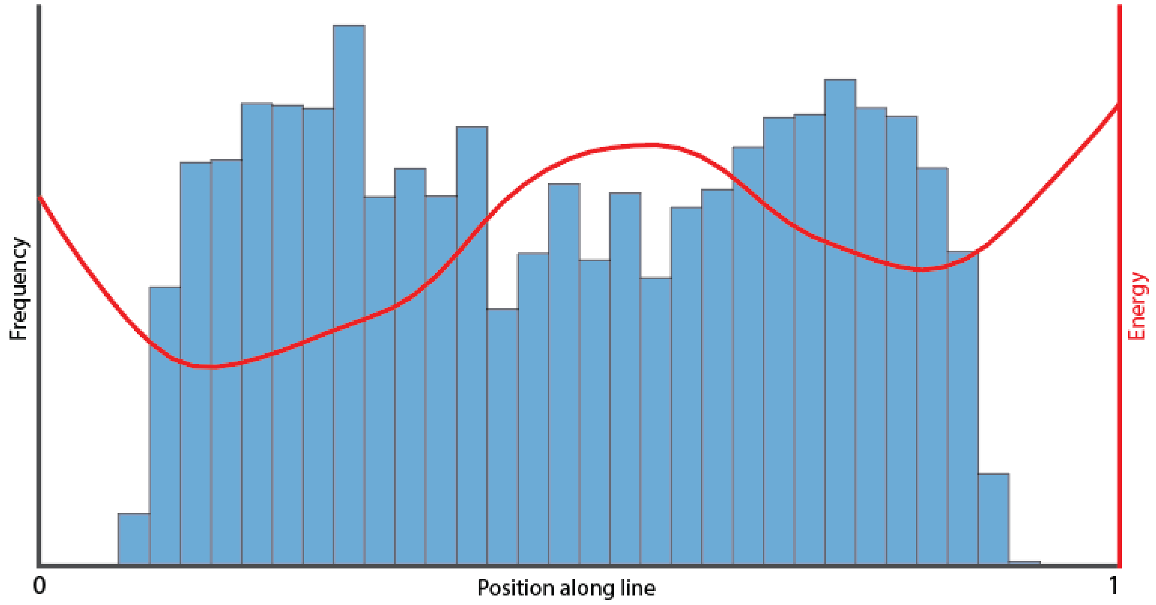

Supplement Figure 3: ***Static model energy landscape.*** *Distribution of all responses is shown in blue. The red line is the energy landscape fit by the static landscape model. Note that the wells of the landscape correspond to increased occupancy in responses.*

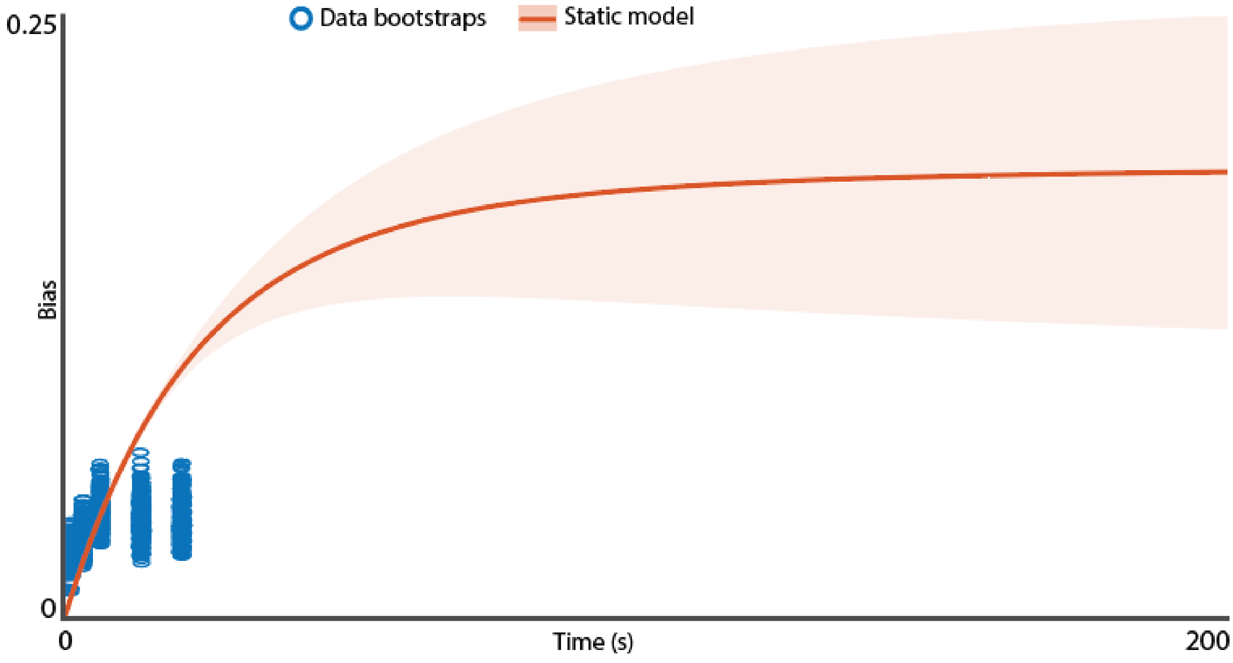

Supplement Figure 4: ***Static model full time course.*** *Simulating the static landscape model for extra time reveals that the bias does eventually saturate albeit at a much higher value than that observed experimentally.*

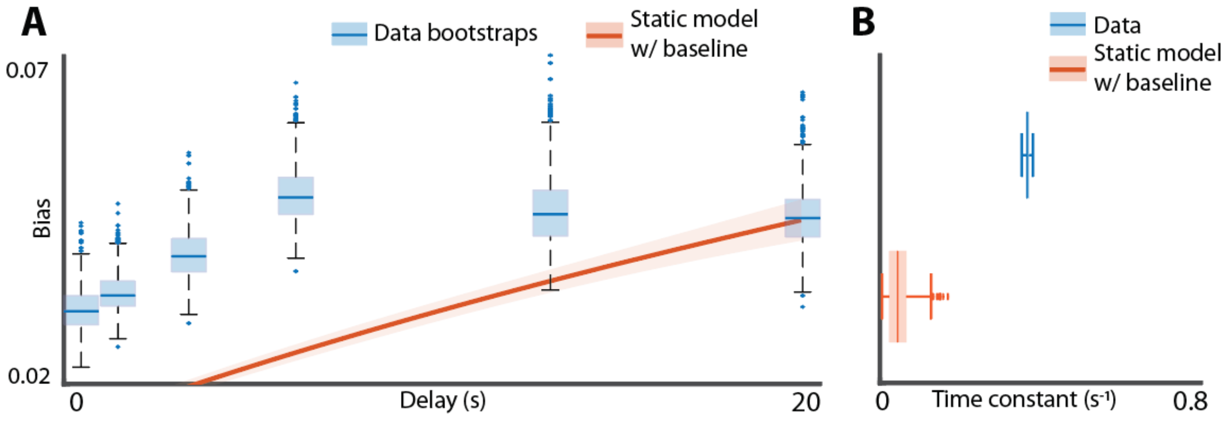

*Supplement Figure 5: **Two stage static model.** The bias strength and the noise amplitude during the initial encoding period were added as additional parameters in the model. **A)** Experimentally observed bias as a function of delay (blue boxes) and the development of bias of a best fitting two stage static model (orange curves, shaded area show 95% confidence intervals) are shown. **B)** Distribution of time constants for the data and the static two stage model are shown.*

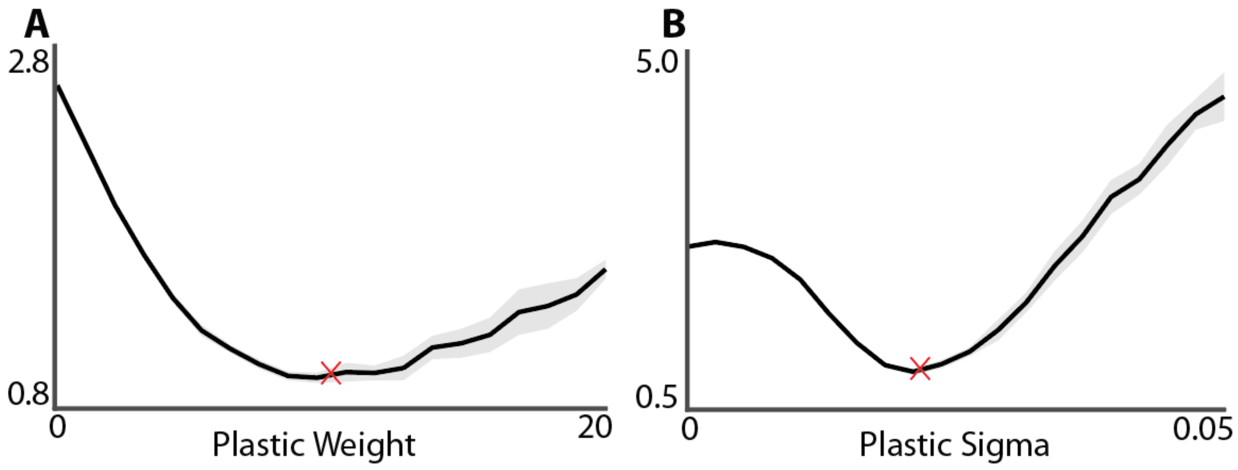

*Supplement Figure 6: **Plastic model parameter robustness**. Plastic model fit captures a local minimum of the model. **A)** Model loss estimated for various values of **p**lasticity sigma β_P. Red X shows the value of the fit parameter in the main text. **B)** Same as **A** for plasticity weight σ_{P}.*

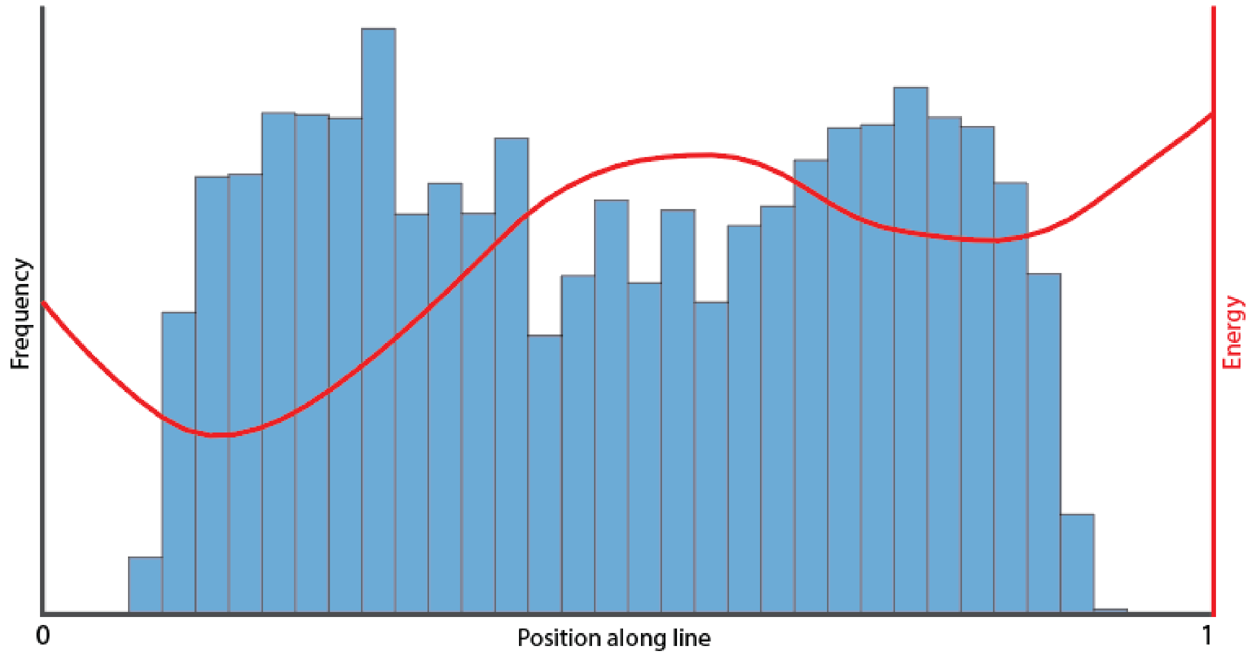

Supplement Figure 7: ***Plastic model energy landscape.*** *Distribution of all subject responses is shown in blue. The red line is the energy landscape fit using the plastic landscape model.*
