# Supplementary material for: Attractor dynamics with activity-dependent plasticity capture human working memory across time scales
Source: Commun Psychol. 2023 Oct 25;1:28. doi: 10.1038/s44271-023-00027-8 (PMC11101211; doi:10.1038/s44271-023-00027-8)
Supplement: Supplementary file 1 [file 44271_2023_27_MOESM3_ESM.pdf]

nature portfolio

Corresponding author(s): *Double-anonymous peer review submissions: write DAPR and your manuscript number here instead of author names.*

Last updated by author(s): *YYYY-MM-DD*

Reporting Summary

Nature Portfolio wishes to improve the reproducibility of the work that we publish. This form provides structure for consistency and transparency in reporting. For further information on Nature Portfolio policies, see our Editorial Policies and the Editorial Policy Checklist.

Statistics

For all statistical analyses, confirm that the following items are present in the figure legend, table legend, main text, or Methods section.

n/a	Confirmed	
☐	☒	The exact sample size (*n*) for each experimental group/condition, given as a discrete number and unit of measurement
☐	☒	A statement on whether measurements were taken from distinct samples or whether the same sample was measured repeatedly
☐	☒	The statistical test(s) used AND whether they are one- or two-sided<br>*Only common tests should be described solely by name; describe more complex techniques in the Methods section.*
☒	☐	A description of all covariates tested
☐	☒	A description of any assumptions or corrections, such as tests of normality and adjustment for multiple comparisons
☒	☐	A full description of the statistical parameters including central tendency (e.g. means) or other basic estimates (e.g. regression coefficient) AND variation (e.g. standard deviation) or associated estimates of uncertainty (e.g. confidence intervals)
☐	☒	For null hypothesis testing, the test statistic (e.g. *F*, *t*, *r*) with confidence intervals, effect sizes, degrees of freedom and *P* value noted<br>*Give P values as exact values whenever suitable.*
☒	☐	For Bayesian analysis, information on the choice of priors and Markov chain Monte Carlo settings
☒	☐	For hierarchical and complex designs, identification of the appropriate level for tests and full reporting of outcomes
☒	☐	Estimates of effect sizes (e.g. Cohen's *d*, Pearson's *r*), indicating how they were calculated

Our web collection on statistics for biologists contains articles on many of the points above.

Software and code

Policy information about availability of computer code

Data collection	The experiment web app was developed in Unity.
Data analysis	The code used to produce the results in this Manuscript can be found at DOI: 10.17605/OSF.IO/FXWMR. Scripts were run in MATLAB 2018a

For manuscripts utilizing custom algorithms or software that are central to the research but not yet described in published literature, software must be made available to editors and reviewers. We strongly encourage code deposition in a community repository (e.g. GitHub). See the Nature Portfolio guidelines for submitting code & software for further information.

Data

Policy information about availability of data

All manuscripts must include a data availability statement. This statement should provide the following information, where applicable:

- Accession codes, unique identifiers, or web links for publicly available datasets
- A description of any restrictions on data availability
- For clinical datasets or third party data, please ensure that the statement adheres to our policy

Data is available at DOI: 10.17605/OSF.IO/FXWMR. No restrictions on sharing. All data is packaged into ParticipantData.rar. Each participant has a separate folder with one JSON file for each 10-block set of trials they completed.

Research involving human participants, their data, or biological material

Policy information about studies with human participants or human data. See also policy information about sex, gender (identity/presentation), and sexual orientation and race, ethnicity and racism.

Reporting on sex and gender	No sex or gender bases analysis was performed.
Reporting on race, ethnicity, or other socially relevant groupings	No analysis on race or ethnicity was performed.
Population characteristics	The average age of participants was 26.2 with a standard deviation of 8.6. Information about the participants' sex was obtained through self-report. There were 86 male participants, and 66 female participants. 9 participants chose not to answer.
Recruitment	161 human participants engaged in an online experiment administered through Prolific. We screened for attention to the task by removing trials with a bias of greater than 0.25 (210 out of 17760 trials). This threshold is set at roughly the 99% quantile of all recorded biases to avoid outlier artifacts. Each participant was asked to complete at least 100 trials at two different delays. They were then given the option to complete more trials at a new set of two delays for increased reward. Data reported in the main text uses the aggregate dataset pooled across participants.
Ethics oversight	All experiments in this study were approved by Institutional Review Board at the University of Pennsylvania and were conducted in accordance with the National Institutes of Health guidelines. All participants gave written informed consent before beginning the experiment.

Note that full information on the approval of the study protocol must also be provided in the manuscript.

Field-specific reporting

Please select the one below that is the best fit for your research. If you are not sure, read the appropriate sections before making your selection.

☒ Life sciences ☐ Behavioural & social sciences ☐ Ecological, evolutionary & environmental sciences

For a reference copy of the document with all sections, see nature.com/documents/nr-reporting-summary-flat.pdf

Life sciences study design

All studies must disclose on these points even when the disclosure is negative.

Sample size	161 human participants engaged in an online experiment administered through Prolific.
Data exclusions	We screened for attention to the task by removing trials with a bias of greater than 0.25 (210 out of 17760 trials). This threshold is set at roughly the 99% quantile of all recorded biases to avoid outlier artifacts. Each participant was asked to complete at least 100 trials at two different delays.
Replication	We did not attempt to replicate the study.
Randomization	We aggregated all data, so randomization is not applicable.
Blinding	We aggregated all data, so blinding is not applicable.

Reporting for specific materials, systems and methods

We require information from authors about some types of materials, experimental systems and methods used in many studies. Here, indicate whether each material, system or method listed is relevant to your study. If you are not sure if a list item applies to your research, read the appropriate section before selecting a response.

Materials & experimental systems

n/a	Involved in the study
☒	☐ Antibodies
☒	☐ Eukaryotic cell lines
☒	☐ Palaeontology and archaeology
☒	☐ Animals and other organisms
☒	☐ Clinical data
☒	☐ Dual use research of concern
☒	☐ Plants

Methods

n/a	Involved in the study
☒	☐ ChIP-seq
☒	☐ Flow cytometry
☒	☐ MRI-based neuroimaging
